# Partial Least Square Model (PLS) as a Tool to Predict the Diffusion of Steroids Across Artificial Membranes

**DOI:** 10.3390/molecules25061387

**Published:** 2020-03-18

**Authors:** Eleni Tsanaktsidou, Christina Karavasili, Constantinos K. Zacharis, Dimitrios G. Fatouros, Catherine K. Markopoulou

**Affiliations:** 1 Laboratory of Pharmaceutical Analysis, Department of Pharmacy, Aristotle University of Thessaloniki, 54124 Thessaloniki, Greece; etsanaktsi@pharm.auth.gr (E.T.); czacharis@pharm.auth.gr (C.K.Z.); 2 Laboratory of Pharmaceutical Technology, Department of Pharmacy, Aristotle University of Thessaloniki, 54124 Thessaloniki, Greece; karavasc@pharm.auth.gr (C.K.); dfatouro@pharm.auth.gr (D.G.F.)

**Keywords:** steroids, Partial Least Squares regression, in vitro permeability, predictive model

## Abstract

One of the most challenging goals in modern pharmaceutical research is to develop models that can predict drugs’ behavior, particularly permeability in human tissues. Since the permeability is closely related to the molecular properties, numerous characteristics are necessary in order to develop a reliable predictive tool. The present study attempts to decode the permeability by correlating the apparent permeability coefficient (P_app_) of 33 steroids with their properties (physicochemical and structural). The P_app_ of the molecules was determined by in vitro experiments and the results were plotted as Y variable on a Partial Least Squares (PLS) model, while 37 pharmacokinetic and structural properties were used as X descriptors. The developed model was subjected to internal validation and it tends to be robust with good predictive potential (R^2^Y = 0.902, RMSEE = 0.00265379, Q^2^Y = 0.722, RMSEP = 0.0077). Based on the results specific properties (logS, logP, logD, PSA and VD_ss_) were proved to be more important than others in terms of drugs P_app_. The models can be utilized to predict the permeability of a new candidate drug avoiding needless animal experiments, as well as time and material consuming experiments.

## 1. Introduction

Steroids are an important category of active pharmaceutical ingredients (APIs). Their structure is characterized by a rigid steroid ring of cyclopentane-perhydro-phenanthrene or sterane [1]. Steroids are small lipophilic molecules and based on their genomic characteristics, they can enter the target cell by a passive diffusion mechanism (mainly by the transcellular route) across plasma membranes [2,3]. As they are derived from cholesterol, they are insoluble in water, and have many pharmacologic effects in almost every major system of the body including the endocrine, cardiovascular, musculoskeletal, nervous, and immune systems [4]. Due to their properties they can be administrated almost through every available administration route such as oral [5], buccal [6,7], transdermal [8], vaginal [9], otic [10], ocular [11], nasal [12], inhalation [13], intravenous [14].

A candidate drug should have appropriate physicochemical and pharmacological properties in order to successfully pass the pre-clinical and clinical trials. Such compound, must exhibit acceptable pharmacokinetic scheme in terms of absorption, distribution, metabolism, excretion and tolerable toxicity (ADMET). The simultaneous optimization of the above processes is one of the main challenges of current pharmacological research [15,16,17]. Unfortunately, these methods are laborious and extremely time-consuming, and they typically require 10–13 years [18,19].

Nowadays, there is a huge number of new candidate drugs that are designed and synthesized in the laboratory. In order to minimize the consumed cost and time, the pharmacokinetic behavior of the compound can be predicted using computational tools (e.g., cheminformatics) providing reliable pharmacokinetic models [15].

Quantitative structure activity/property relationship (QSAR/QSPR) studies correlate the physicochemical properties of a compound to biological activity [16]. Such studies have been extensively used for developing predictive models in which the chemical structures and biological properties are correlated. Alternately, such data could be obtained through in vitro, ex vivo and in vivo experiments [15].

Due to development of cheminformatics, there are plenty of QSAR modeling techniques, such as support vector machine (SVM), artificial neural networks (ANNs), multiple linear regression (MLR), principal component analysis (PCA) and partial least squares (PLS) regression [15]. The PLS method provides the possibility for linear correlation of numerous observations and multiple X variables with one or more Y variables [17].

Generally, PLS is a rapid and effective method for developing robust and reliable QSAR models. It has been widely used for the design of plenty of predictive patterns, such as for the placental-barrier permeability [18], blood–brain-barrier permeability from simulated chromatographic conditions [19], central nervous system (CNS) drug exposure [20], blood–brain barrier permeation of α-adrenergic and imidazoline receptor ligands using the parallel artificial membrane permeability assay (PAMPA) technique [21]. Additionally, PLS tool was used to discover potent Wee1 inhibitors [22], to evaluate 2-cyano-pyrimidine analogs as cathepsin-K inhibitors [23] and also to characterize the performance of dry powder inhalers [24].

The main scope of this research is to develop a new model that would be able to predict the permeability of a compound having the chemical structure of steroids. This approach is based on the correlation of its characteristics (physicochemical and structural properties) with the permeability of the molecule determined by in vitro experiments. In the present study the permeability of 33 steroids has been investigated using vertical Franz type diffusion cells including a synthetic cellulose membrane as model membrane [25]. Due to low water solubility of steroids, solubility enhancers (e.g., Polyethylene Glycol and Polysorbate 80) were used in order to achieve the desirable concentration for each compound. The obtained experimental results were treated using the Partial Least Squares (PLS) methodology. The developed models were validated and were found to be statistically significant with good predictive ability.

## 2. Results

### 2.1. Partial Least Squares (PLS) Methodology

#### 2.1.1. Dataset Compilation

The present study involves the data processing of the derived experimental results using the PLS methodology. A Soft Independent Modeling of Class Analogies Simca-P (version 9; Umetrics, Uppsala, Sweden) [26,27] chemometric software was used to construct the classical PLS models.

The object of the research was to investigate the effect of several properties of steroids on their permeability at a hydrophilic cellulose membrane. The number of models developed in this process was five since the Y response variable was either calculated differently, or refers to four separate sampling times, after the first hour of the experiments. Therefore, *P_2h_, P_4h_, P_6h_, P_8h_* models denote the number of steroids permeating the artificial membrane at 2 h, 4 h, 6 h and 8 h, respectively (Y variable: permeability μg/cm^2^), whereas *model P_app_* expresses the (Y) variable calculated as the *apparent permeability* factor. In the present study, the theoretical explanation of steroids permeability was mainly based on model *P_app_,* which is considered as the most important. Each of the five models contained 32 observations (analytes which belong to steroids) with 46 X variables and one Y variable. The large amount of X variables used was considered necessary, even though some of them were proved to be of minor interest. In order to implement the proposed models, it was rather important to carefully collect and record some of their most important properties and structural characteristics. Each dataset consists of three parts. The first is the column containing the observations (33 analytes). The second is the main part of each dataset and it is populated by a few physicochemical and structural characteristics of the analytes. There are 37 descriptors (physicochemical properties), which were calculated using a series of different software or free online databases (Table 1).

In more details, the studied compounds were imported in the free cheminformatics program Data Warrior [28], in order to predict the clogP (calculated partition coefficient, log(C_octanol_/C_water_)), the clogS (water solubility at 25 °C, log mol/L), the number of hydrogen bond acceptors and donors, the number of aromatic rings, carboxyl groups, carbonyl groups, hydroxyl groups, also the molecular complexity, the total surface area (Å^2^), the relative polar surface area (Å^2^), the polar surface area—PSA (Å^2^), the shape index, the molecular flexibility and the drug-likeness. Descriptors related with the pharmacokinetic properties of the compounds were calculated by inserting the simplified molecular-input line-entry system (SMILES) of the drugs in the freely accessible web server pkCSM [29]. The pharmacokinetic properties employed were Caco2 permeability (log P_app_), intestinal absorption (% absorption), skin permeability (log K_p_), steady state of volume distribution (VD_ss_, log L/kg), blood-brain barrier (BBB) permeability (logBB), CNS permeability (logPS), and total clearance (log mL/min/kg).

The melting point, °C of the compounds was obtained from Open Melting Point Dataset [30] and also from EPA DSSTox [31]. The topological polar surface area (Å^2^) [32] was also predicted from PubChem data [33]. Moreover, Marvin, a free ChemAxon tool [34] was used in order to draw and characterize chemical structures of the compounds for the calculation of pK_a_, logP, number of rings, distribution coefficient (logD) at pH 7.4 and their water solubility at 25 °C (logS, log mol/L). Details about the molar volume V_m_ (cm^3^), molar refractivity (cm^3^), PSA (Å^2^), polarizability (cm^3^), molar volume (cm^3^) were obtained via ACD/Labs [35]. All the above descriptors represented the X variables of the model developed and they are summarized in Table 1.

It is important to mention that some descriptors (e.g., logP) were calculated using more than one software program since there was a need to confirm their dominant role in the model. The structural features were found in the constitutional parameters and are outlined with nine descriptors used to decode the chemical structure of the analytes on the same basis. This was achieved by using integer numbers and zero to indicate the presence, the multiplicity or the absence of a structural characteristic.

The third part of the PLS dataset is a column with Y variable that corresponds to the calculated permeability of the drugs on Franz cells experiments. The Y variable is expressed as apparent permeability *P_app_*, or permeability *P_2h_. P_4h_. P_6h_. P_8h_* at different sampling times (2h, 4h, 6h, 8h).

Variables’ Importance in the Projection (VIP) column plots provide information about the importance of the parameters in the dataset. However, apart from the importance of a descriptor in a model, it is crucial to know whether its impact on the signal response is positive or negative. For this purpose, it was necessary to evaluate the loadings plots (w × c[1]/w × c[2]) of the models at the first two components.

#### 2.1.2. Validation

Normalization of the observations (values of both X and Y variables) was achieved using mean centering and unit variance scaling. Validation of the PLS models was performed making use of three techniques, Cross-Validation (CV) the external and the internal validation [26,36].

**Figure 1:**
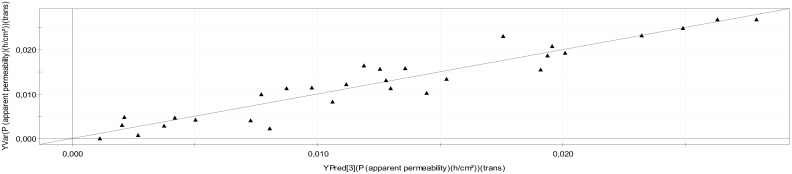
Observed versus estimated values of model *P_app_* with *apparent permeability* values as (*Y*) variable, RMSEE = 0.00265379.

First, the Cross Validation (CV) was achieved by dividing data into seven parts and each 1/7th of samples was excluded to build a model with the remaining 6/7th of samples. The Y values for the excluded data were then predicted by this new model and the procedure was repeated until all samples had been predicted once. If the original model is valid, then the plot of predicted Y versus actual measured Y values will be a straight line with the RMSEE (Root Mean Squares Error of Estimation) as low as possible (Figure 1) and calculated from Equation (1).

(1) $$RMSEE=\sqrt[{}]{\frac{\sum {\left(\overset{\wedge }{y_{i}}-y_{i}\right)}^{2}}{N}}$$

*(ŷ_i_ represents the estimated Papp value for the i^th^ object and y_i_ the reference P_app_ value)*

The prediction error sum of squares (PRESS) is a good measure of the predictive power of the model, providing information about the significance of the component (a component is considered significant when PRESS/Residual sum of squares < 1). Using the appropriate number of significant components, the total models were fit (Table 2) according to Haaland and Thomas criteria [37].

**Figure 2:**
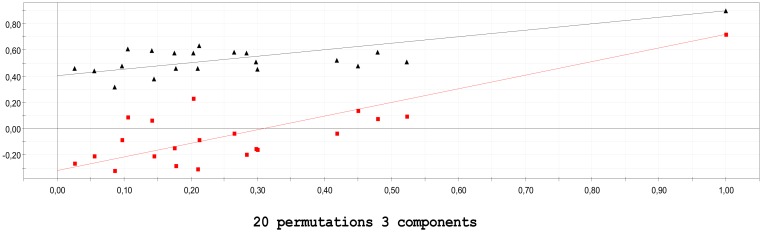
Internal validation test with 20 permutations, for model *P_app_*. Intercepts: R^2^ = (0.0, 0.405) marked black, Q^2^ = (0.0, −0.32) marked red.

Verification of the reliability of the models was also achieved with the response permutation methodology (internal validation). During this process, the data for Y are not changed but they are randomly rearranged. Then the PLS model is applied again on the modified Y data and the R^2^Y and Q^2^Y values are recalculated. The above are compared with the initial values providing a first indication about the validity of the model. This process is repeated (20 permutations/model) and the results represent the statistical evaluation of the significance of the R^2^Y and Q^2^Y parameters in the initial model. In the diagram derived, the y-axis represents the R^2^Y/Q^2^Y values of all models and the x-axis represents the correlation coefficient between the modified and initial responses. In order to summarize the results of the method, regression analysis is applied on both R^2^Y and Q^2^Y and the regression lines are obtained. Verification of statistical significance of the original assessments is in accordance with the intercept limits regarding permutations (Figure 2) and they are set to R^2^Y < 0.3−0.4 and Q^2^Y < 0.05 [38].

External validation was performed dividing data set of model *P_app_* in two equal parts training and test set. Thereafter, the calculation of the training set and the prediction of the test were completed, and their roles were swapped. The quality of external prediction was assessed by the Q^2^ (Q^2^_train_ = 75.4, Q^2^_test_ = 71.5) and the Root Mean Square Error of Prediction (RMSEP) from Equation (2) value, where RMSEP was equal to 0.00770361 for the training set and 0.00764925 for the test set, respectively.

(2) $$RMSEP=\sqrt{\frac{\sum {\left(obs-pred\right)}^{2}}{N}}$$

The fact that the two estimates are similar means that these two subsets have similar information and can be combined in a total data set. External prediction may also aim the model to predict the Y values of new entities, in other words, entities excluded from the data set. Hence, the model is applicable either to interpret the behavior of a steroid based on its physicochemical properties or to predict the behavior of an unknown drug in the human body. PLS regression analysis is appropriate since it uses linear correlations and at the same time can predict with high reliability.

### 2.2. Interpretation of Steroids Permeability Through PLS

**Figure 3:**
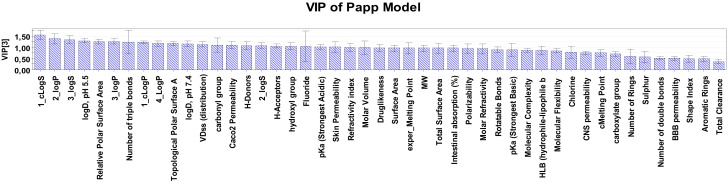
Variables’ Importance in the Projection (VIP) plot for the *apparent permeability P* values of model P, at 95% confidence level.

The permeability of a group of steroids across an artificial membrane was estimated using a hydrophilic cellulose membrane and the apparent permeability coefficient values were calculated. The mainly PLS model *P_app_* was established using 32 compounds and a 47-descriptor analysis aimed at identification of the most critical molecular properties that influence permeability across the artificial membrane. According to the VIP plot of *P_app_* model (Figure 3) logS, logP, logD (at pH 5.5 and 7.4), PSA (topological and relative) and VD_ss_ were found to be the most influential descriptors (VIP > 1) on the apparent permeability of the tested steroids through the cellulose membrane. All the other descriptors were found to have a similar and non-discriminating effect on the permeability of the tested compounds (VIP < 1).

**Figure 4:**
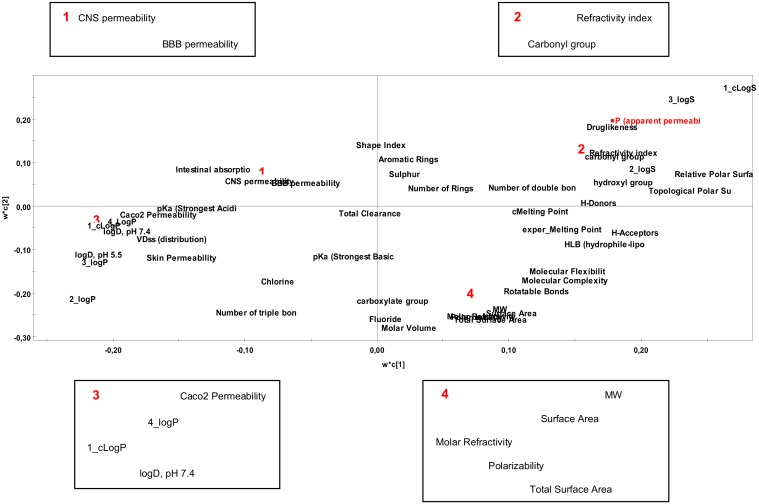
A scatter *w × c[1]* versus *w × c[2]* plot for *P_app_* model.

Further information on the positive or negative effect of the X variables on the permeability is derived from scatter *w* × *c[1]* versus *w* × *c[2]* plot for P_app_ model in Figure 4.

Drug dissolution is almost always a precondition for adequate permeability and absorption and, therefore, poor aqueous solubility is commonly associated with limited drug bioavailability [39]. It has been also exemplified that poor solubility may originate from high lipophilicity, resulting in poor permeability [40]. Compliant to this consensus, the findings of the current study support the positive contribution of logS (marked red in Table 3) and the respective negative effect of logP (marked blue in Table 3) on the P_app_ of the tested steroids.

PSA has been recently recognized as a useful predictor of permeability. It defines the polar part of a molecule and correlates with passive molecular transport through membranes. It has been previously observed that compounds with PSA < 60 Å are highly permeable, in contrast to those with PSA > 120 Å that are poorly permeable [41]. In that context, optimal permeability has been recognized when PSA is below 120 Å. Apart from prednisolone 21-sodium succinate (PSA = 141 Å), which has been classified as an outlier, PSA values for all steroids evaluated in the present study were below the cutoff value (PSA < 110 Å) suggested for the identification of poorly permeable compounds. Even though it’s been recognized that lower PSA contributes to higher permeability [41], that trend was not confirmed here, mainly due to the absence of extreme variations in the PSA values and considering the relatively narrow range of PSA for the tested steroids.

Lipophilicity is considered one of the main factors with a positive effect on drug permeation across biological membranes. However, an inverse relationship between logP and permeability may be encountered upon increasing drug lipophilicity, due to a greater tendency for drug partitioning from the aqueous phase to the membrane [42]. It has been previously proposed that steroid permeation through a cellulose acetate membrane is a sequence of adsorption and desorption events with an intermittent membrane diffusion process, with the latter being dependent on the permeant’s molecular size, its interaction with the membrane and the membrane’s structural characteristics [43]. Such interaction might be favored with decrease in steroid polarity because, despite its hydrophilic nature, cellulose acetate remains more hydrophobic relative to the water [43]. Even though molecular size and polarity (with the latter being typically expressed as PSA or H-donors and acceptors) have been adversely associated with drug permeation [44,45], a positive correlation between steroids’ polarity and permeability has been previously recognized. In particular, among three oestrogens of similar molecular size and distinctive polarities, an increase in permeability was observed with decreasing steroid-membrane interactions [46]. An inverse correlation between clogP and steroid permeability across Caco-2 cell monolayers was also recognized by Faasen et al., [47]. The results demonstrated a faster diffusion of the more hydrophilic steroids across the cell monolayers compared to the more hydrophobic ones. These findings coincide with the findings of the current study, concluding that steroids with lower logP gravitate towards a higher permeability.

Volume of distribution at steady state (VD_ss_) is rendered as a solid indicator of drug distribution in the body reflecting its ability to permeate membranes and bind in tissues. Certain criteria have been defined to discriminate between drugs with high and low VD_ss_. LogP has been shown to be a significant determinant of VD_ss_, which along with the presence of Cl atoms and molecule compactness, have a positive contribution on this descriptor, while polarity and strong electrophiles have a negative contribution on VD_ss_ [48]. High VD_ss_ values (> 42 L), representative of more lipophilic drugs, indicate a high likeliness of drug distribution throughout body tissues, whereas low VD_ss_ values (< 3 L) associate with a predominant location in the systemic circulation [49]. According to the findings of the current study, a negative correlation between P_app_ and VD_ss_ was obtained, which aligns with the positive correlation between logP and VD_ss_ also observed in the present study.

**Figure 5:**
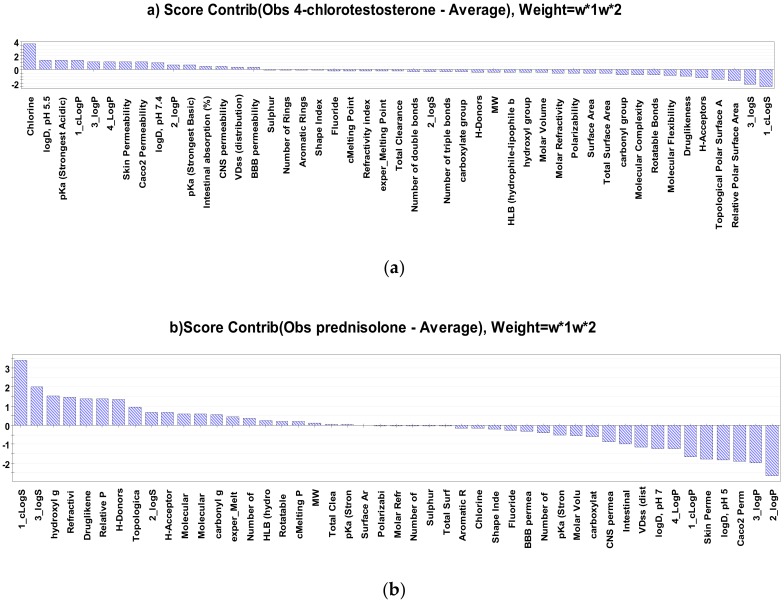

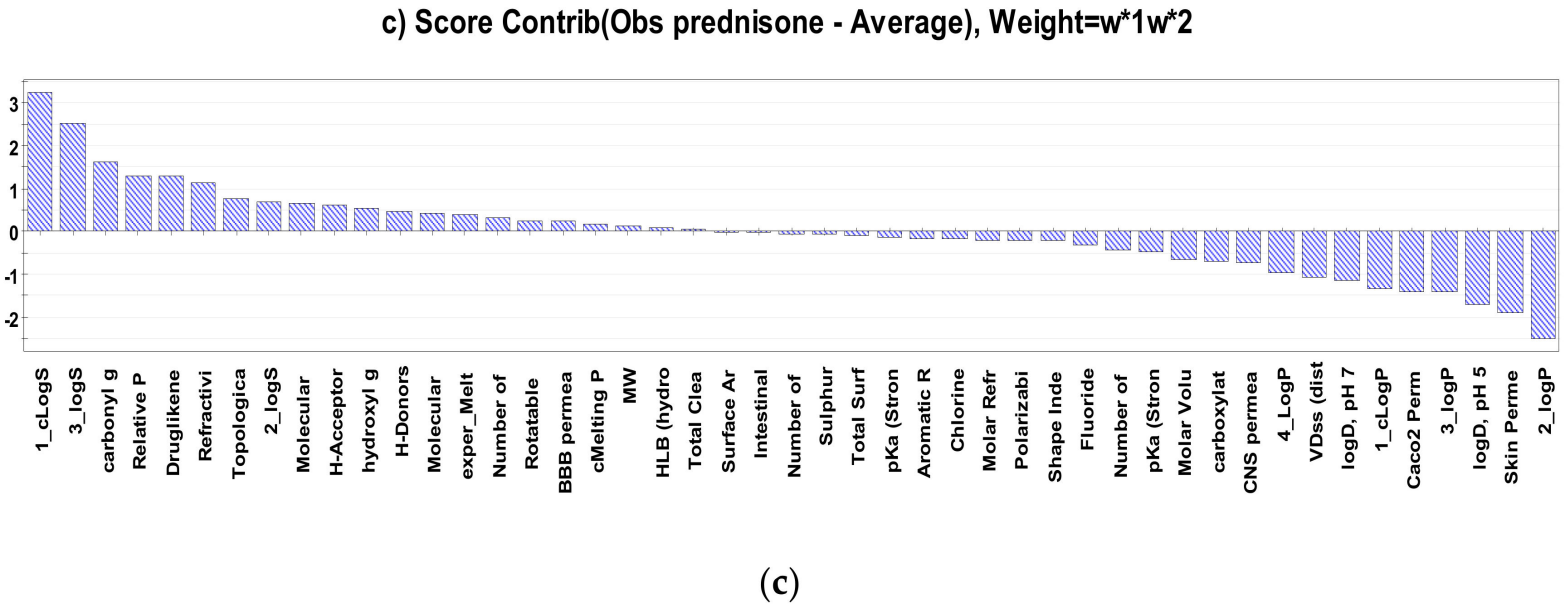
Contribution score plot of (**a**) 4-chlorotestosterone, (**b**) prednisolone and (**c**) prednisone, versus the remaining observations.

Among the steroids evaluated, 4-chlorotestosterone demonstrated the lowest and prednisone and prednisolone the highest *P_app_* value. As illustrated in Figure 5a, the presence of the Cl atom seems to be the most determinant descriptor affecting *P_app_* of 4-chlorotestosterone. The chlorine atom as substituent in a molecule has been shown to increase its lipophilicity [19,50]. This positive contribution of Cl atoms to logP justifies the decrease in the apparent permeability of 4-chlorotestosterone, due to the negative correlation between logP and *P_app_*, as already demonstrated in the present study. On the other hand, for both steroids showing the highest P_app_, a combination of the same descriptors (logP and logS) was identified to be the most discriminative (Figure 5b,c). The steroids with the highest aqueous solubility and the lowest lipophilicity tend to diffuse faster across the cellulose membrane, compared to the most hydrophobic and less soluble steroids, which tend to a lower permeability.

Additionally, androstanolone was considered as outlier during the first 4 h of the in vitro permeability study, showing significantly higher *P_app_* values compared to the rest of the tested steroids. Based on its contribution plot at both 2 h and 4 h, it is evident that a combination of parameters related to the molecular size of androstanolone (number of double bonds, shape index, molar refractivity, polarizability, MW) are lower than the respective average values of the tested compounds, whereas pK_a_ was found to be higher compared to the average pK_a_ values of the tested steroids. Since all steroids remain unionized in the pH used in the current study, the contribution of pK_a_ to *P_ap_*_p_ may be considered negligible. On the other hand, results signify the importance of molecular size on *P_app_* with an inverse relationship existing between the two.

As already mentioned, drug diffusion across membranes consists of a series of events including drug transfer from the hydrophilic aqueous environment of the donor compartment, through the more hydrophobic (relative to the water) membrane to the hydrophilic aqueous environment of the receptor compartment. The ease of drug diffusion may be explained by elucidating the most significant parameters affecting drug permeability in a time-dependent manner. As seen in Table 3, the most critical descriptors affecting the amount of drug permeated at 2 h mainly relate to the molecular size of the permeants including shape index, MW and molar volume which is also directly related to the refractivity index and polarizability of the steroids tested [19], all the above are marked green in Table 3.

All these parameters contribute negatively to drug permeation, which could translate to hindering drug diffusion to the receptor phase and, instead, increasing their retention and affinity towards the membrane. This trend seems to change with time, with logS (red marked on Table 3) and logP (blue marked on Table 3) being the dominant descriptors affecting drug permeation thereafter.

The utility of in silico models in predicting drug permeability across biological membranes has been recognized as a time- and cost-efficient tool to facilitate drug discovery and development. The PLS model has been previously employed to identify the most critical molecular parameters affecting the permeability and retention of 17b-carboxamide steroids across an artificial membrane (parallel artificial membrane permeability assay (PAMPA)), as a means to predict their permeability across human skin [41]. In another study, Zhang et al., (2015) confirmed the good predictability of the PLS model, highlighting its potential utility as a high-throughput screening tool of placental drug permeability [18]. PLS and the genetic algorithm-PLS method have also been found appropriate in identifying the optimal subset of descriptors that have a significant contribution on drugs’ permeability across Caco-2 cell monolayers [51], as well as on in vivo human drug intestinal permeability [52].

The present study is initially considered to be a reliable tool for the development of a theoretical background that will explain the permeability of steroids to biological membranes. In addition, the remarkable ability of PLS models to predict the behavior of drugs increases the usefulness of the proposed technique in designing new more effective steroids.

## 3. Materials and Methods

### 3.1. Reagents, Materials, Solutions

Acetonitrile (ACN) and water (HPLC grade) were purchased from VWR Chemicals (Radnor, USA), and Sigma-Aldrich (Darmstadt, Germany) respectively. For LC-MS analyses, water and ACN were both LC-MS gradient grade and provided by Sigma-Aldrich (Darmstadt, Germany).

Phosphate buffer saline (PBS) pH 7.4 was prepared by dissolving sodium chloride (8.0 g), sodium phosphate dibasic (1.44 g), and potassium phosphate monobasic (0.24 g), (Merck, Darmstadt, Germany) and potassium chloride (0.20 g) (Chem-Lab nv, Zedelgem, Belgium) in 1 L of distilled water. Polyethylene glycol (PEG 200) obtained by Sigma-Aldrich (Darmstadt, Germany) and polysorbate 80 (tween 80) provided by ManisChemicals (Athens, Greece).

The dialysis tubing cellulose membrane (flat width 43 mm) was obtained from Sigma-Aldrich (Darmstadt, Germany). The corticosteroids substances (Table 4) were United Stated Pharmacopeia—USP grade and were obtained from Sigma-Aldrich (Darmstadt, Germany).

### 3.2. Methods

#### 3.2.1. Solubility Study

Solubility studies were carried out for the most lipophilic corticosteroid based on its logS value, obtained from Marvin (COMP 9, logS = −5.79, 25 °C). The study was conducted in PBS (pH 7.4) in the presence of polyethylene glycol 200 (PEG 200) and polysobrate 80 (Tween80) used as co-solvents at different ratios, owing the ability to enhance the water solubility of lipophilic drugs. In detail, an excess amount of the drug was added in the above-mentioned solvent mixtures and sonicated for 1 h at 30 °C. Then, the mixtures were kept under mild agitation for 48 h at room temperature to facilitate the dissolution. Any visible remaining drug particulates were removed by centrifugation at 2000× *g* for 20 min. The supernatants were quantified by HPLC analysis using the conditions described in Table 5. Based on the results of the solubility study, PBS, 40 % (*w*/*w*) PEG 200 and 0.2 % (*w*/*w*) Tween 80 was used as the solvent mixture. The same procedure was followed for all compounds in this solvent mixture and a final concentration of 100 μg/mL was selected for the in vitro permeation studies.

#### 3.2.2. In Vitro Permeation Studies

Cellulose membrane was properly treated and mounted in the Franz diffusion cells (diffusion area 4.9 cm^2^, compartment volume 20 mL). The acceptor compartment was filled with PBS pH 7.4 and the donor compartment was filled with 1 mL of the formulation described above (100 μg/mL of the compounds). Permeation studies were conducted under constant stirring (90 rpm) at 37 °C. Samples of 0.5 mL were withdrawn from the acceptor compartment at predetermined time intervals (30 min, 1 h, 2 h, 4 h, 6 h, 8 h) and replaced with fresh and preheated PBS. Experiments were repeated in triplicates for each compound and blank experiments containing only the medium were also performed. The samples were analyzed by HPLC without any previous pretreatment.

Steady state flux (*J_ss_*) was calculated from the slope of the linear section of the plot of the amount of permeated compound per unit area (μg/cm^2^) against to time. The apparent permeability coefficient (*P_app_*) was calculated using Equation (3), where *C_d_* is the initial concentration of the drug in the donor compartment and *J_ss_* is the steady state flux.

(3) $$P_{app}=\frac{J_{ss}}{C_{d}}\;$$

#### 3.2.3. HPLC Experimental Conditions/Method Validation

The drug content was quantified using either the HPLC-UV (High-performance Liquid Chromatography–Ultraviolet) or LC-MS (Liquid Chromatography–Mass Spectrometry) instrument. The HPLC-UV setup was equipped with two LC-20AD pumps, a SIL-20AC HT auto-sampler, a CTO-20AC column oven and an SPD-M20A diode array detector (Shimadzu). For LC-MS analysis, a Shimadzu LC-MS 2020 single-quadrupole mass spectrometer with an electrospray ion source (ESI) was utilized. A nitrogen gas generator N_2_LCMS (Nitrogen Generator, Claind) was used throughout in this study. The temperature of the curved dessolvation line was set at 250 °C, the N_2_ nebulizer gas flow was maintained at 1.5 L/min and the drying gas flow was set at 15 L/min, while the interface voltage was set at 4.5 kV in positive mode. The analytical column temperature was kept constant at 30 °C. The stationary phase was a C_18_ column (4.6 × 50 mm, 2.5 μm, Shimadzu). The sample injection volume was 5 μL in all cases. The mobile phase was a binary mixture of acetonitrile and water at appropriate ratio for each compound in order to avoid the prolonged analysis time. The HPLC-UV and LC-MS experimental conditions used for the analysis of each compound are described in Table 5.

Both the HPLC-UV and LC-MS methods were validated in-house according to ICH (International Conference Harmonization) guidelines [53]. The calibration curves for each compound was linear (*r*^2^ > 0.999) in the range of LOQ-20 μg/mL (six calibration levels). Regression analysis, LOD (Limit of Detection) and LOQ (Limit of Quantification) values were tabulated in Table 6. Samples were analyzed in triplicate.

## 4. Conclusions

An attempt to describe, experimentally and theoretically, the ability of a drug to permeate human tissues and be distributed in the body was carried out. For this purpose, five different PLS regression models were applied, using the permeability factor *P_app_* as Y variable, for a series of steroids/drugs versus their physicochemical and structural properties (X variables). The determination of P_app_ factor was performed by in vitro drug permeability experiments across a cellulose membrane. According to the VIP values of the *P_app_* model, the two factors with the stronger effect were logS and logP, which are dominant to the phenomenon with reverse influence. It is also remarkable that the permeability of steroids is dependent on the effect of numerous parameters and cannot be considered as a result of a specific factor (physicochemical property or structural feature). Finally, it is worth noting that one of steroids (4-chlorotestosterone) with chloro-substituted moiety did not penetrate the membrane at all, which makes it unique.

The PLS model seems to accurately describe this simulation and predict with reliability the behavior for an unknown drug. Based on such databases, researchers could use the information provided to predict whether a drug can be distributed in a tissue via passive transfer.

## Figures and Tables

**Table 1:** X Descriptors of Dataset.

Open Melting Point Dataset	EPA DSSTox	Data Warrior	ACD/Labs	Marvin	PubChem	pkCSM
Melting Point		1_cLogP	2_logP	3_logP	Topological PSA	MW
		1_cLogS	logD, pH 7.4	2_logS		4_LogP
		Hydrogen Bond Acceptors	Refractivity index	pKa (Strongest Acidic)		3_logS
		Hydrogen Bond Donors	Molar Refractivity	pKa (Strongest Basic)		Double bonds
		Aromatic Rings	PSA	HLB		Rotatable Bonds
		Carboxyl group	Polarizability	N_o_ of Rings		Surface Area
		Carbonyl group	Molar Volume			Caco2 Permeability
		Hydroxyl group				Intestinal absorption
		Total Surface Area				log Kp
		Relative PSA				VD_ss_
		PSA				log BB
		Shape Index				logPS
		Molecular Complexity				Total Clearance
		Molecular Flexibility				
		Drug-likeness				

**Table 2:** Statistical Parameters in Partial Least Squares (PLS) regression models.

Models	R^2^Y ^1^	Q^2^ (cum) ^2^	Number of Components	Excluded Observations
P_app_	0.902	0.722	3	3
P_2h_	0.802	0.567	3	1
P_4h_	0.847	0.656	3	2
P_6h_	0.846	0.659	3	2
P_8h_	0.872	0.605	3	3

^1^$R^{2}=\sum_{i=1}^{N}{\left(\hat{y_{i}}-y_{i}\right)}^{2}/\sum_{i=1}^{N}{\left(y_{i}-\bar{y_{i}}\right)}^{2}$ ($\bar{y_{i}}$ represents the means of the true P_app_ values in the predictor set). ^2^ $Q^{2}=\frac{1-\mathrm{PRESS}}{\mathrm{SumSquares}}$.

**Table 3:** Models’ VIP values.

Models’ VIP Values							
P_2h_		P_4h_		P_6h_		P_8h_	
Var ID (Primary)	M2.VIP[3]	Var ID (Primary)	M3.VIP[3]	Var ID (Primary)	M3.VIP[3]	Var ID (Primary)	M3.VIP[3]
Total Clearance	1.62825	1_cLogS ^1^	1.41597	1_cLogS	1.48033	1_cLogS	1.47640
Shape Index ^2^	1.45142	Shape Index	1.31645	logD, pH 5.5 ^3^	1.19879	No. of triple bonds	1.27167
Molar Volume	1.27798	Molar Volume	1.21737	3_logP	1.19154	Chlorine	1.22264
cMelting Point	1.25731	cMelting Point	1.18929	3_logS	1.18122	3_logS	1.18501
Refractivity index	1.21565	Molar Refractivity	1.15522	2_logS	1.17849	3_logP	1.17937
1_cLogS	1.21432	logD, pH 5.5	1.15097	Molar Volume	1.17612	Molar Volume	1.17567
Molar Refractivity	1.19812	3_logS	1.15097	Drug-likeness	1.16409	Drug-likeness	1.17289
Polarizability	1.17929	Polarizability	1.14560	cMelting Point	1.14712	Fluoride	1.16495
Total Surface Area	1.15973	2_logS	1.14311	Chlorine	1.13175	logD, pH 5.5	1.16166
No of triple bonds	1.14692	Chlorine	1.14308	Fluoride	1.12684	2_logS	1.15902
2_logS	1.12114	carboxylate group	1.13474	Molar Refractivity	1.12456	cMelting Point	1.13450
carboxylate group	1.11557	No of triple bonds	1.12947	Polarizability	1.11544	Molar Refractivity	1.11508
MW	1.09348	Fluoride	1.12462	1_cLogP	1.11174	Polarizability	1.10855
H-Donors	1.09223	Total Surface Area	1.12262	logD, pH 7.4	1.10117	Refractivity index	1.10584
Chlorine	1.08855	3_logP	1.12234	No of triple bonds	1.09925	Total Surface Area	1.08631
3_logS	1.07715	Refractivity index	1.10689	Total Surface Area	1.09019	1_cLogP	1.08316
logD, pH 5.5	1.05540	exper_Melting Point	1.07645	4_LogP	1.08878	hydroxyl group	1.07759
Rotatable Bonds	1.05147	MW	1.06777	Refractivity index	1.08517	4_LogP	1.06769
Surface Area	1.04603	H-Donors	1.05761	2_logP	1.08068	H-Donors	1.06767
exper_Melting Point	1.03503	logD, pH 7.4	1.05533	hydroxyl group	1.06590	logD, pH 7.4	1.06241
hydroxyl group	1.03056	1_cLogP	1.05320	H-Donors	1.06502	2_logP	1.0599
logD, pH 7.4	1.00956	hydroxyl group	1.04762	MW	1.04701	exper_Melting Point	1.05272
3_logP	0.97296	2_logP	1.04431	exper_Melting Point	1.03706	MW	1.04095
Caco2 Permeability	0.96943	Surface Area	1.04168	carboxylate group	1.03300	Surface Area	1.02086
4_LogP	0.96517	4_LogP	1.03678	Surface Area	1.02096	Shape Index	0.99368
1_cLogP	0.95059	Druglikeness	1.01070	Shape Index	0.99572	Relative PSA	0.98728
2_logP	0.93301	Rotatable Bonds	0.98079	Relative PSA	0.98111	carboxylate group	0.97549
H-Acceptors	0.88952	Caco2 Permeability	0.96573	Caco2 Permeability	0.96947	Rotatable Bonds	0.95868

^1^ red indicates positive contribution, ^2^ green indicates size related descriptors, ^3^ blue indicates negative contribution.

**Table 4:** Structures of studied compounds.

Steroids Structures													
	Compound	Double Bonds	C^2^	C^3^	C^4^	C^5^	C^7^	C^9^	C^10^	C^11^	C^13^	C^16^	C^17^
17a-hydroxyprogesterone	COMP 1	4 = 5							CH_3_		CH_3_		COCH_3_, OH
4-chlorotestosterone	COMP 2	4 = 5		=O	Cl				CH_3_		CH_3_		OCOCH_3_
Androstanolone	COMP 3								CH_3_		CH_3_		OH
Betamethasone dipropionate	COMP 4	1 = 2, 4 = 5		=O				F	CH_3_	OH	CH_3_	CH_3_	COCH_2_ OCOC_2_H_5_, OCOC_2_H_5_
Betamethasone valerate	COMP 5	1 = 2, 4 = 5		=O				F	CH_3_	OH	CH_3_	CH_3_	COCH_2_OH, OCOC_4_H_9_
Budesonide	COMP 6	1 = 2, 4 = 5		=O					CH_3_	OH		a	a, COCCH_2_OH
Cortisone acetate	COMP 7	4 = 5		=O					CH_3_	=O	CH_3_		COCH_2_OCOCH_3_, OH
Dehydro-isoandrosterone	COMP 8	5 = 6		OH					CH_3_		CH_3_		=O
Deoxycorticosterone acetate	COMP 9	4 = 5		=O					CH_3_		CH_3_		COCH_2_OCOCH_3_
Dexamethasone	COMP 10	1 = 2, 4 = 5		=O				F	CH_3_	OH	CH_3_	CH_3_	CCOCH_2_OH, OH
D-norgestrel	COMP 11	4 = 5		=O							CH_2_CH_3_		C≡CH, OH
Estriol	COMP 12	1 = 2, 3 = 4, 5 = 10		OH								OH	OH
Estrone	COMP 13	2 = 3, 4 = 5, 10 = 1		OH							CH_3_		=O
Ethinylestradiol	COMP 14	1 = 2, 3 = 4, 5 = 10		OH							CH_3_		C≡CH, OH
Ethisterone	COMP 15	4 = 5		=O							CH_3_	CH_3_	C≡CH, OH
Fludrocortisone acetate	COMP 16	4 = 5		=O				F	CH_3_	OH	CH_3_		COCH _2_OCOCH_3_, OH
Formebolone	COMP 17	1 = 2, 4 = 5	CHO	=O					CH_3_	OH	CH_3_		CH_3_, OH
Hydrocortisone	COMP 18	4 = 5		=O					CH_3_	OH	CH_3_		COCH_2_OH, OH
Hydrocortisone acetate	COMP 19	4 = 5		=O					CH_3_	OH	CH_3_		COCH_2_OCOCH_3_, OH
Medroxyprogesterone acetate	COMP 20	4 = 5		=O		CH_3_			CH_3_		CH_3_		OCOCH_3_, COCH_3_
Methandriol	COMP 21	5 = 6		OH									CH_3_, OH
Methyl testosterone	COMP 22	4 = 5		=O					CH_3_		CH_3_		CH_3_, OH
Norethisterone	COMP 23	4 = 5		=O							CH_3_		C≡CH, OH
Prednisolone	COMP 24	1 = 2, 4 = 5		=O					CH_3_	OH	CH_3_		COCH_2_OH, OH
Prednisolone 21-sodium succinate	COMP 25	1 = 2, 4 = 5		=O					CH_3_	OH	CH_3_		COCH_2_OCOCH_2_CH_2_OCOH, OH
Prednisolone acetate	COMP 26	1 = 2, 4 = 5		=O					CH_3_	OH	CH_3_		COCH_2_OCOCH_3_, OH
Prednisone	COMP 27	1 = 2, 4 = 5		=O					CH_3_	=O	CH_3_		COCH_2_OH, OH
Progesterone	COMP 28	4 = 5		=O					CH_3_		CH_3_		COCH_3_
Spironolactone	COMP 29	4 = 5		=O			SCOCH_3_		CH_3_		CH_3_		b
Testosterone	COMP 30	4 = 5		=O					CH_3_		CH_3_		OH
Testosterone acetate	COMP 31	4 = 5		=O					CH_3_		CH_3_		OCOCH_3_
Testosterone propionate	COMP 32	4 = 5		=O					CH_3_		CH_3_		OCOC_2_H_5_
trans-Androsterone	COMP 33			OH					CH_3_		CH_3_		=O
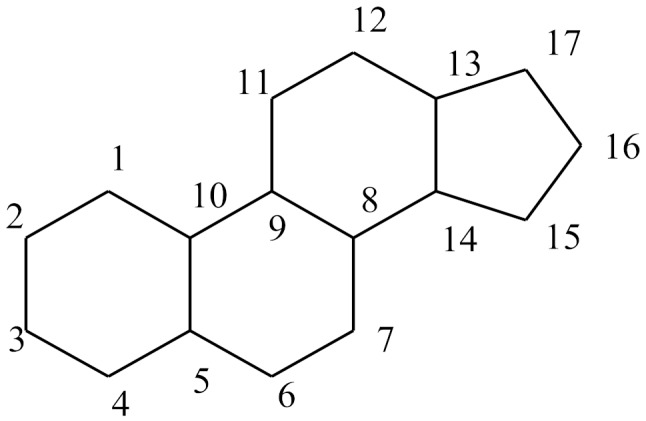 sterane					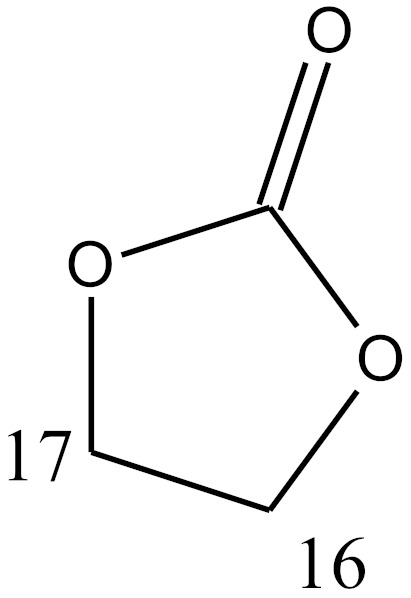 a						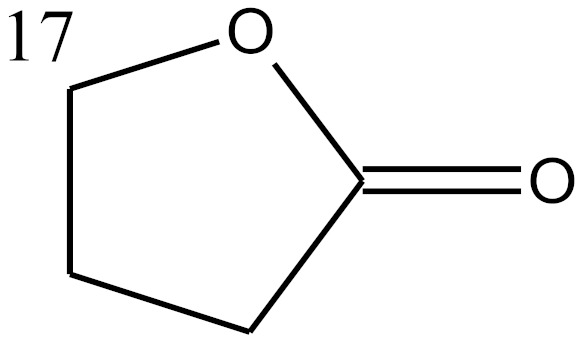 b		

**Table 5:** Chromatographic Conditions.

Compound	Detector	Flow (mL/min)	Retention Factor (*k*’)	λ (nm)	Quantification Ion ^2^ (m/z)
COMP 1	DAD ^1^	0.3	1.11	240	-
COMP 2	MS ^3^	0.5	4.67	-	365 [(M+CH_3_CN)+H]^+^
COMP 3	MS	0.5	4.06	-	332 [(M+CH_3_CN)+H]^+^
COMP 4	DAD	0.3	2.18	240	-
COMP 5	DAD	0.5	1.45	240	-
COMP 6	DAD	0.5	1.93	240	-
COMP 7	DAD	0.4	3.05	240	-
COMP 8	DAD	0.4	2.55	230	-
COMP 9	DAD	0.4	2.43	240	-
COMP 10	DAD	0.4	2.46	240	-
COMP 11	DAD	0.4	3.34	240	-
COMP 12	DAD	0.5	1.49	205	-
COMP 13	DAD	0.5	3.98	205	-
COMP 14	DAD	0.4	0.92	240	-
COMP 15	DAD	0.3	2.56	205	-
COMP 16	DAD	0.4	3.12	240	-
COMP 17	DAD	0.4	2.58	220	-
COMP 18	DAD	0.3	0.83	240	-
COMP 19	DAD	0.5	0.96	240	-
COMP 20	DAD	0.5	0.74	240	-
COMP 21	MS	0.5	4.76	-	287 [(M-H_2_O)+H]^+^
COMP 22	DAD	0.5	0.87	240	-
COMP 23	DAD	0.4	2.48	240	-
COMP 24	DAD	0.4	2.18	240	-
COMP 25	DAD	0.4	3.11	244	-
COMP 26	DAD	0.4	2.43	240	-
COMP 27	DAD	0.4	2.45	240	-
COMP 28	DAD	0.5	0.87	240	-
COMP 29	DAD	0.5	4.04	238	-
COMP 30	DAD	0.5	0.67	240	-
COMP 31	DAD	0.5	1.12	240	-
COMP 32	DAD	0.5	1.66	240	-
COMP 33	MS	0.5	4.50	-	373 [(M+2CH_3_CN)+H]^+^

^1^ DAD: diode array detector, ^2^ performed at single ion monitoring (SIM) mode, ^3^ MS: mass spectrometry.

**Table 6:** Analytical figures of merit of HPLC-UV and LC-MS methods.

Compound	R ^2^	Intercept	Slope	LOD ^1^ (μg/mL)	LOQ ^2^ (μg/mL)
COMP 1	0.9997	1123	43133	0.01	0.04
COMP 2	0.9947	−5470	38034	0.27	0.90
COMP 3	0.9997	1252	16203	0.05	0.18
COMP 4	0.9997	−9501	28376	0.11	0.38
COMP 5	0.9998	−4479	20526	0.05	0.18
COMP 6	0.9998	552	16428	0.04	0.12
COMP 7	0.9996	−3723	28562	0.03	0.12
COMP 8	0.9997	−993	4454	0.32	1.07
COMP 9	0.9993	−4166	26523	0.02	0.06
COMP 10	0.9990	−11577	13079	0.18	0.60
COMP 11	0.9998	−12551	43421	0.01	0.03
COMP 12	1.0000	−1090	51548	0.07	0.24
COMP 13	0.9996	−3671	51988	0.06	0.19
COMP 14	0.9990	26763	79974	0.01	0.04
COMP 15	1.0000	706	30294	0.01	0.03
COMP 16	0.9999	7399	25115	0.04	0.14
COMP 17	0.9992	12588	45247	0.05	0.18
COMP 18	0.9997	−6597	62213	0.01	0.03
COMP 19	0.9985	26809	19890	0.03	0.10
COMP 20	1.0000	802	37460	0.01	0.05
COMP 21	0.9996	436	10500	0.05	0.16
COMP 22	0.9999	798	25856	0.02	0.05
COMP 23	0.9999	−162	38688	0.01	0.04
COMP 24	0.9999	1568	38840	0.03	0.10
COMP 25	1.0000	−353	13103	0.04	0.12
COMP 26	0.9997	−2111	22329	0.04	0.13
COMP 27	0.9997	−256	26564	0.04	0.14
COMP 28	0.9997	916	32027	0.01	0.04
COMP 29	1.0000	−1564	26756	0.02	0.06
COMP 30	1.0000	3599	113379	0.01	0.03
COMP 31	1.0000	6794	25375	0.01	0.04
COMP 32	1.0000	11227	54069	0.01	0.02
COMP 33	0.9971	−303	5860	0.03	0.10

^1^ LOD: based on S/N = 3 criteria, ^2^ LOQ: based on S/N = 10 criteria.

## References

[1] Alexander K., July G.D., Hugh M. (2010). Structure and Nomenclature of Steroids. Steroid Analysis.

[2] Giorgi E.P., Stein W.D. (1981). The transport of steroids into animal cells in culture. Endocrinology.

[3] Oren I., Fleishman S.J., Kessel A., Ben-Tal N. (2004). Free diffusion of steroid hormones across biomembranes: A simplex search with implicit solvent model calculations. Biophys. J..

[4] McKay C.J., Kufe D.W., Pollock R.E., Weichselbaum R.R. (2003). Pharmacologic Effects of Corticosteroids. Holland-Frei Cancer Medicine.

[5] Kuhnz W. (1990). Pharmacokinetics of the contraceptive steroids levonorgestrel and gestodene after single and multiple oral administration to women. Am. J. Obstet. Gynecol..

[6] Nair M., Chien Y.W. (1993). Buccal delivery of progestational steroids: I. Characterization of barrier properties and effect of penetrant hydrophilicity. Int. J. Pharm..

[7] Gass M., Rebar R.W., Cuffie-Jackson C., Cedars M.I., Lobo R.A., Shoupe D., Judd H.L., Buyalos R.P., Clisham P.R. (2004). A short study in the treatment of hot flashes with buccal administration of 17-β estradiol. Maturitas.

[8] Badoud F., Boccard J., Schweizer C., Pralong F., Saugy M., Baume N. (2013). Profiling of steroid metabolites after transdermal and oral administration of testosterone by ultra-high pressure liquid chromatography coupled to quadrupole time-of-flight mass spectrometry. J. Steroid Biochem. Mol. Biol..

[9] Hassan A.S., Soliman G.M., El-Mahdy M.M., El-Gindy G.E.-D.A. (2017). Solubilization and enhancement of ex vivo vaginal delivery of progesterone using solid dispersions, inclusion complexes and micellar solubilization. Curr. Drug Deliv..

[10] Creber N.J., Eastwood H.T., Hampson A.J., Tan J., O’Leary S.J. (2019). Adjuvant agents enhance round window membrane permeability to dexamethasone and modulate basal to apical cochlear gradients. Eur. J. Pharm. Sci..

[11] Xu X., Sun L., Zhou L., Cheng Y., Cao F. (2020). Functional chitosan oligosaccharide nanomicelles for topical ocular drug delivery of dexamethasone. Carbohydr. Polym..

[12] Guennoun R., Fréchou M., Gaignard P., Lière P., Slama A., Schumacher M., Denier C., Mattern C. (2019). Intranasal administration of progesterone: A potential efficient route of delivery for cerebroprotection after acute brain injuries. Neuropharmacology.

[13] Demirca B.P., Cagan H., Kiykim A., Arig U., Arpa M., Tulunay A., Ozen A., Aydıner E.K., Baris S., Barlan I. (2015). Nebulized fluticasone propionate, a viable alternative to systemic route in the management of childhood moderate asthma attack: A double-blind, double-dummy study. Respir. Med..

[14] Zhuo X., Huang X., Yan M., Li H., Li Y., Rong X., Lin J., Cai J., Xie F., Xu Y. (2019). Comparison between high-dose and low-dose intravenous methylprednisolone therapy in patients with brain necrosis after radiotherapy for nasopharyngeal carcinoma. Radiother. Oncol..

[15] Ferreira L.L.G., Andricopulo A.D. (2019). ADMET modeling approaches in drug discovery. Drug Discov. Today.

[16] Brown E.W. (1962). © 1962 Nature Publishing Group. Nat. Int. J. Sci..

[17] Wold S., Sjöström M., Eriksson L. (2001). PLS-regression: A basic tool of chemometrics. Chemom. Intell. Lab. Syst..

[18] Zhang Y.H., Xia Z.N., Yan L., Liu S.S. (2015). Prediction of placental barrier permeability: A model based on partial least squares variable selection procedure. Molecules.

[19] Kouskoura M.G., Piteni A.I., Markopoulou C.K. (2019). A new descriptor via bio-mimetic chromatography and modeling for the blood brain barrier (Part II). J. Pharm. Biomed. Anal..

[20] Bergström C.A.S., Charman S.A., Nicolazzo J.A. (2012). Computational prediction of CNS drug exposure based on a novel in vivo dataset. Pharm. Res..

[21] Vucicevic J., Nikolic K., Dobričić V., Agbaba D. (2015). Prediction of blood-brain barrier permeation of α-adrenergic and imidazoline receptor ligands using PAMPA technique and quantitative-structure permeability relationship analysis. Eur. J. Pharm. Sci..

[22] Hu Y., Zhou L., Zhu X., Dai D., Bao Y., Qiu Y. (2019). Pharmacophore modeling, multiple docking, and molecular dynamics studies on Wee1 kinase inhibitors. J. Biomol. Struct. Dyn..

[23] Wang J., Peng W., Li X., Fan W., Wei D., Wu B., Fan L., Wu C., Li L. (2019). Towards to potential 2-cyano-pyrimidines cathepsin-K inhibitors: An in silico design and screening research based on comprehensive application of quantitative structure-activity relationships, molecular docking and ADMET prediction. J. Mol. Struct..

[24] Elia A., Cocchi M., Cottini C., Riolo D., Cafiero C., Bosi R., Lutero E. (2016). Multivariate data analysis to assess dry powder inhalers performance from powder properties. Powder Technol..

[25] Ng S., Rouse J.J., Sanderson F.D., Eccleston G.M. (2012). The Relevance of Polymeric Synthetic Membranes in Topical Formulation Assessment and Drug Diffusion Study. Arch. Pharm. Res..

[26] Umetrics (2001). Simca-P 9.0—User Guide and Tutorial.

[27] Wu Z., Li D., Meng J., Wang H., Vinzi V.E., Chin W.W., Henseler J., Wang H. (2010). Introduction to SIMCA-P and Its Application. Handbook of Partial Least Squares: Concepts, Methods and Applications.

[28] Sander T., Freyss J., von Korff M., Rufener C. (2015). DataWarrior: An open-source program for chemistry aware data visualization and analysis. J. Chem. Inf. Model..

[29] Pires D.E.V., Blundell T.L., Ascher D.B. (2015). pkCSM: Predicting small-molecule pharmacokinetic and toxicity properties using graph-based signatures. J. Med. Chem..

[30] Bradley J.-C., Lang A., Williams A. (2014). Jean-Claude Bradley Double Plus Good (Highly Curated and Validated) Melting Point Dataset. Figshare.

[31] Williams A.J., Grulke C., Edwards J., McEachran A.D., Mansouri K., Baker N., Patlewicz G., Shah I., Wambaugh J., Judson R.S. (2017). The CompTox Chemistry Dashboard: A community data resource for environmental chemistry. J. Cheminform..

[32] Ertl P., Rohde B., Selzer P. (2000). Fast calculation of molecular polar surface area as a sum of fragment-based contributions and its application to the prediction of drug transport properties. J. Med. Chem..

[33] Kim S., Chen J., Cheng T., Gindulyte A., He J., He S., Li Q., Shoemaker B.A., Thiessen P., Yu B. (2018). PubChem 2019 update: Improved access to chemical data. Nucleic Acids Res..

[34] (2012). ChemAxon, Marvin. https://chemaxon.com/.

[35] ACD/Labs (2015). Advanced Chemistry Development Inc. https://www.acdlabs.com/index.php.

[36] Lapinsh M., Prusis P., Uhlén S., Wikberg J.E.S. (2005). Improved approach for proteochemometrics modeling: Application to organic compound—Amine G protein-coupled receptor interactions. Bioinformatics.

[37] Haaland D.M., Thomas E.V. (1988). Partial least-squares methods for spectral analyses. 1. Relation to other quantitative calibration methods and the extraction of qualitative information. Anal. Chem..

[38] Lapins M., Eklund M., Spjuth O., Prusis P., Wikberg J.E.S. (2008). Proteochemometric modeling of HIV protease susceptibility. BMC Bioinform..

[39] Dahan A., Miller J.M. (2012). The Solubility-permeability interplay and its implications in formulation design and development for poorly soluble drugs. AAPS J..

[40] Tan N.C., Yu P., Kwon Y.-U., Kodadek T. (2008). High-throughput evaluation of relative cell permeability between peptoids and peptides. Bioorg. Med. Chem..

[41] Chi C.T., Lee M.H., Weng C.F., Leong M.K. (2019). In silico prediction of PAMPA effective permeability using a two-QSAR approach. Int. J. Mol. Sci..

[42] Boyd B.J., Bergström C.A., Vinarov Z., Kuentz M., Brouwers J., Augustijns P., Brandl M., Bernkop-Schnürch A., Shrestha N., Préat V. (2019). Successful oral delivery of poorly water-soluble drugs both depends on the intraluminal behavior of drugs and of appropriate advanced drug delivery systems. Eur. J. Pharm. Sci..

[43] Barry B.W., El Eini D.I.D. (1976). Influence of non-ionic surfactants on permeation of hydrocortisone, dexamethasone, testosterone and progesterone across cellulose acetate membrane. J. Pharm. Pharmacol..

[44] Palm K., Stenberg P., Luthman K., Artursson P. (1997). Polar molecular surface properties predict the intestinal absorption of drugs in humans. Pharm. Res..

[45] Veber D.F., Johnson S.R., Cheng H.-Y., Smith B.R., Ward K.W., Kopple K.D. (2002). Molecular properties that influence the oral bioavailability of drug candidates. J. Med. Chem..

[46] Barry B.W., Brace A.R. (1977). Permeation of oestrone, oestradiol, oestriol and dexamethasone across cellulose acetate membrane. J. Pharm. Pharmacol..

[47] Faassen F., Kelder J., Lenders J., Onderwater R., Vromans H. (2003). Physicochemical Properties and Transport of Steroids across Caco-2 Cells. Pharm. Res..

[48] Zhivkova Z., Mandova T., Doytchinova I. (2015). Quantitative structure—Pharmacokinetics relationships analysis of basic drugs: Volume of distribution. J. Pharm. Pharm. Sci..

[49] Smith D.A., Beaumont K., Maurer T.S., Di L. (2015). Volume of distribution in drug design. J. Med. Chem..

[50] Naumann K. (2000). Influence of chlorine substituents on biological activity of chemicals: A review. Pest. Manag. Sci..

[51] Yamashita F., Wanchana S., Hashida M. (2002). Quantitative structure/property relationship analysis of Caco-2 permeability using a genetic algorithm-based partial least squares method. J. Pharm. Sci..

[52] Winiwarter S., Ax F., Lennernäs H., Hallberg A., Pettersson C., Karlén A. (2003). Hydrogen bonding descriptors in the prediction of human in vivo intestinal permeability. J. Mol. Graph. Model..

[53] ICH (2005). ICH Topic Q2 (R1) Validation of Analytical Procedures: Text and Methodology.

